# The mediation of health-promoting lifestyle on self-perceived health status and quality of life among nurses: a cross-sectional study

**DOI:** 10.1186/s12912-023-01608-y

**Published:** 2023-11-28

**Authors:** Kuei-Ying Wang, Ching-Ming Chien, Huan-Fang Lee, Yohana Yobelina

**Affiliations:** 1 Department of Nursing, College of Health Sciences, Chang Jung Christian University, No.1, Changda Rd., Gueiren District, Tainan City, 711301 Taiwan (Republic of China); 2 Department of Medical Science Industries, College of Health Sciences, Chang Jung Christian University, No.1, Changda Rd., Gueiren District, Tainan City, 711301 Taiwan (Republic of China); 3 Department of Nursing, College of Medicine, National Cheng Kung University, No.1, University Road, Tainan City, 701 Taiwan (Republic of China); 4 Department of Translation and Interpretation Studies, College of Humanities and Social Sciences, Chang Jung Christian University, No.1, Changda Rd., Gueiren District, Tainan City, 711301 Taiwan (Republic of China)

**Keywords:** Self-perceived health, Quality of life, Health-promoting lifestyle, Mediation, Nurse

## Abstract

**Background:**

Nurses with busy workloads lack the time to maintain health, leading to a decline in physical and mental health and quality of life. It is widely accepted that self-perception of health triggers health-promoting behaviors and impacts the quality of life; however, the relationship between these factors among nurses is unclear. The purpose of this study was to investigate the ability of a health-promoting lifestyle to mediate the relationship between self-perceived health and quality of life among nurses.

**Methods:**

A cross-sectional survey was conducted in four regional Taiwanese teaching hospitals with over 500 beds. The survey used stratified random sampling of 600 nurses who had worked for more than six months. The Self-Perceived Health Questionnaire, the Health-Promoting Lifestyle Profile, and the World Health Organization Quality of Life Scale were used to measure nurses’ self-perceived health (SPH), health-promoting lifestyle (HPL), and quality of life (QoL). A Hayes PROCESS analysis and bootstrapping method were used for the mediation analysis.

**Results:**

A total of 518 nurses’ data was included in the analysis. Nurses perceived their health status as less favorable than their colleagues, but frequently adopted health promotion behaviors. Nurses reported a moderate QoL. QoL and SPH were correlated (r = .33) and a high correlation between QoL and HPL (r = .64) was found. SPH and HPL both affect QoL (B = 0.077 and 0.070). SPH and HPL explained 42.6% of the variation in QoL. HPL played a partial mediation role.

**Conclusions:**

The study confirmed that HPL has an important role in mediating nurses’ SPH and QoL. Nurse administrators are advised to encourage nurses to monitor their health status and provide health promotion mechanisms to improve their quality of life.

## Background

Health care is a high-pressure profession, and the pressure on healthcare workers comes not only from the work itself, but also from patients, colleagues, and hospital systems [1]. A systematic review study demonstrated that work stress, even when it originates from an intrinsic component such as overcommitment, can increase the risk of adverse health conditions [2]. Previous studies identified an association between rotating shifts and an increased risk of cardiometabolic and gastrointestinal diseases, as well as cancer-related mortality among nurses who work in a hospital [3–5].

In Taiwan, nurses face additional work stress that is primarily associated with heavy workloads. The nurse-patient ratio ranges from 1:7 to 1: 12 during the day, 1:12 to 1:20 in the evening and 1:15 to 1:30 during the night shift [6]. This has led to poor perceived health among Taiwanese nurses. A study investigated nurses’ self-perceived health (SPH), a person’s overall evaluation of their own health [7]—and discovered only 12.4% of nurses rated their health as very good, while 16.5% perceived poor health [6].

A national survey in 2006 reported that 33% of nurses working in hospitals experienced work stress [8]. Nurses are constantly faced with stressful situations that cause physical and emotional exhaustion, which may lead to a lower quality of life [9, 10]. The World Health Organization defines quality of life (QoL) as a person’s overall sense of well-being, including physical health, psychological state, level of independence, social relationships, personal beliefs and relationships to salient features [11]. Nurses who worked night shifts in particular experienced poorer sleep quality than those who worked day shifts [12, 13]. Beyond the physiological effects, night shifts can lead to behavioral and psychological consequences [14]. It’s noteworthy that many hospital nurses tend to adopt unhealthy lifestyles, including low physical activity levels and poor dietary behaviors [15]. This issue is particularly relevant in Taiwan, where a high prevalence of low physical activity among females is observed [16]. In regard to this, research has emphasized health promotion lifestyle (HPL) as a major strategy to help individuals take actions that could benefit their health [17].

Several studies have been conducted to investigate the relationship between HPL, SPH, and QoL. Studies examining the impacts of COVID-19 on US adults, including a change in their lifestyle characteristics and well-being, revealed positive associations between perceived health and healthy lifestyles, as well as the detrimental effect of a poor HPL on overall QoL [18, 19]. There is also compelling evidence linking HPL with a higher QoL [20, 21]. Chiang et al. suggested that adopting an HPL can lead to positive health outcomes, affecting work satisfaction, mental health, and overall quality of life [22]. Although some studies have shown partial mediation effects of HPL on SPH and QoL, others have revealed full mediation effects [23–25]. This mediating effect of a lifestyle offers valuable insight into potential strategies to enhance the quality of life of nurses and other populations.

Nurses are an essential part of the health care system, and their health-promoting lifestyle can influence the beliefs or attitudes towards health promotion. When people perceive their own health, they can adopt health promotion behaviors to reduce adverse health problems and improve their quality of life [26]. However, few research has been conducted on nurses concerning this phenomenon. The role of HPL in mediating nurses’ SPH and QoL remains unclear. Therefore, the purpose of this study was to investigate the ability of a health-promoting lifestyle to mediate the relationship between self-perceived health and quality of life among nurses.

## Methods

### Design and participants

A cross-sectional survey method was used for this descriptive correlational study. A total of 600 questionnaires were distributed across four regional teaching hospitals, each representing the northern, southern, eastern, and western parts of Taiwan. Stratified random sampling was carried out based on nursing competency levels one through four, with level 1 representing the most junior and level 4 representing the most senior. The inclusion criteria for this study include full-time nurses with an RN credential and at least 6 months of work experience. This duration guarantees that nurses have gained the required competence to perform their duties safely and successfully without direct supervision, including the capacity to work night shifts. The study focused on RNs and charge nurses who participated actively in clinical practice, including outpatient clinics, emergency departments, operating rooms, critical care units, and general wards. Part-time nurses and nursing administrators were excluded from the scope of the study.

### Measurement

Socio-demographic were included in the data collection: age, education level, marital status, number of children, total years of work experience, job title, monthly income.

Self-perceived health status was assessed using a scale modified by Hung [27] based on one of the subscales in The Medical Outcome Study 36-Item Short-Form Health Survey (MOS SF-36): general health perceptions [28]. This modification allowed for a more focused evaluation of participants’ general health perceptions while using the reliable framework of the MOS SF-36. Comprising five elements, SPH was measured using a five-point Likert scale ranging from 1 ‘very poor’ to 5 “very good”. The total scores range from 5 to 25, where a higher score indicates a better self-perception of health. The Cronbach’s alpha was 0.87 in Hung’s study [27] and 0.90 in the current study.

The health-promoting lifestyle was measured using the Taiwan version of the Health Promoting Lifestyle Profile (HPLP) [29] originally developed by Walker et al. in 1987 [30]. This version of the scale is well established in the Taiwanese context and widely adopted to assess health-promoting lifestyle and behaviors [31, 32]. The 40-item instrument includes six dimensions: self-actualization, health responsibility, exercise, nutrition, interpersonal support, and stress management. HPLP uses a 4-point Likert scale ranging from 1 ‘never’ to 4 “always”, for a total score range of 40 to 160. A higher score reflects a healthier lifestyle. A Cronbach’s alpha of 0.92 was reported by Chen et al. [29] study and was 0.92 in the current study.

The quality of life was measured using the World Health Organization Quality of Life Scale Brief Version of Taiwan (WHOQOL-BREF Taiwan Version), as developed following a standard translation procedure and validity verifications [33]. The WHOQOL-BREF Taiwan version was selected due to its relevance and suitability for Taiwanese study populations. It was designed with 2 additional items to account for Taiwanese cultural adaptations, which resulted in a total of 28 items. 26 items covered four dimensions of physical health, mental health, social relationships, and environment, and two items were general questions: “How would you rate your quality of life in general?“ and “Overall, are you satisfied with your health?“. The answers were measured through a 5-point Likert scale ranging from “1” representing “never” to “5” representing “always”. The total score ranges from 28 to 140, with a higher score indicating better QoL. A Cronbach’s alpha of 0.91 was reported by Yao et al. [33], and 0.93 in the current study.

### Procedure

After approval was obtained from the Institutional Review Board of each medical center, the researcher visited the Director of Nursing of each facility to explain the purpose and procedures and obtained permission. A trained research assistant contacted the employed nurses and distributed the consent forms and questionnaires. All participants signed their informed consent and completed the questionnaire. These forms were to be returned to the researchers within one month using the provided envelope.

### Data analysis

Data analyses for hypothesis testing adopted Hayes’ PROCESS regression (SPSS WIN 25.0 program with PROCESS macro 3.5 version). The characteristics and instrument scores were described using descriptive statistics. The correlations between the continuous variables were analyzed using Pearson’s correlation coefficients. To test the mediation of health-promoting lifestyle, the PROCESS macro (5,000 bootstrap resamples) was used with Model 4. A significant mediating effect is indicated when the 95% CI does not contain 0. Covariates (age, education level, years of working, employment type, and job title) were controlled for, and the study variables were standardized.

## Results

### Sample characteristics and scores of quality of life (QoL)

Of the 600 questionnaires distributed, 536 nurses provided data, resulting in a response rate of 89.3%. There were 18 incomplete questionnaires, leaving only 518 in total for data analysis. The majority of nurses were under 30 years old (41.7%, n = 216), held a university degree (70.3%, n = 364), had no children (62.5%, n = 324), and reported 4 to 12 years of work experience (37.6%, n = 195). The majority were direct care nurses (87.8%, n = 455). Total mean QoL scores were significantly different by age (group over 40 > 21–30 and 31–40, *p* = .03), years of work experience (group over 12 > 1–4 and 4–12, *p* = .001) and nursing position (Charge nurse > Direct care nurse, *p* = .008) (Table 1).

**Table 1:** Characteristics of the subjects and scores on quality of life (N = 518)

Variable	Frequency	Percentage	Mean	SD	*p*
Age (years)^a^					0.03
21–30	216	41.7	3.4	0.53	
31–40	197	38.0	3.4	0.54	
> 40	105	20.3	3.6	0.45	
Education					0.105
Under college	154	29.7	3.4	0.50	
Above university	364	70.3	3.5	0.53	
Marital status					0.843
Single	292	56.4	3.5	0.53	
Married	226	43.6	3.5	0.51	
Children					0.625
No	324	62.5	3.5	0.52	
Yes	194	37.5	3.5	0.52	
Working experience (years)^b^					0.001
① 1–4	179	34.6	3.4	0.51	③ > ① & ②
② 4–12	195	37.6	3.4	0.56	
③ > 12	144	27.8	3.6	0.44	
Nursing position					0.008
Direct care nurse	455	87.8	3.4	0.52	
Charge nurse	63	12.2	3.6	0.49	

^a^The post hoc analysis was shown that in the age variable, group over 40 years has higher scores than group 21–30 years and group 31–40 years in QoL scores

^b^The post hoc analysis was shown that in the working experience variable, group over 12 years has higher scores than group 1–4 years and group 4–12 years in QoL scores

### Nurse perception of self-perceived health (SPH), health-promoting lifestyle (HPL), and quality of life (QoL)

The total mean score for self-perceived health status was 2.8 ± 0.79. The total mean score of the health-promoting lifestyle was 2.6 ± 0.44; Physical activity scored the lowest (2.2 ± 0.61), and interpersonal support scored the highest (3 ± 0.61). The study revealed that nurses almost often adopted health promotion behaviors. The mean total QoL score was 3.5 ± 0.52. Physical health scored the lowest (3.4 ± 0.46), and mental health scored the highest (3.5 ± 0.57). The overall quality of life was moderate for the nurses in this study (Table 2).

**Table 2:** The mean scores of nurses on SPH, HPL, and QoL (n = 518)

Variable	Mean		SD
Self-Perceived Health (SPH)	2.8		0.79
Health Promoting Lifestyle (HPL)	2.6		0.44
Self-Actualization	2.8		0.52
Health Responsibility	2.5		0.56
Stress Management	2.7		0.58
Interpersonal Support	3.0		0.61
Nutrition	2.6		0.63
Physical Activity	2.2		0.61
Quality of life (QoL)	3.5		0.52
Physical Health	3.4		0.46
Psychological State	3.5		0.57
Social Relationship	3.5		0.65
Environment	3.5		0.71

### Relationship between self-perceived health (SPH), health-promoting lifestyle (HPL) and quality of life (QoL)

The findings showed that there was a moderate positive correlation between QoL and SPH (r = .330, p < .001). Furthermore, a strong positive correlation was found between QoL and HPL (r = .639, p < .001). Among the HPL dimensions, self-actualization was found to have the highest correlation with all dimensions of quality of life (r between 0.45 and 0.60), while physical activity had the lowest correlation (r between 0.312 and 0.381) (see Table 3). These results confirmed that, among nurses, self-perceived health is positively related to both health-promoting lifestyle and quality of life. In addition, a health-promoting lifestyle was found to have a significant positive correlation with quality of life.

**Table 3:** Correlation between scores of instruments

	HPL	SA	HR	SM	IS	Nutrition	PA	QOL	PH	PS	SR	Environment
Self-Perceived Health (SPH)	0.362	0.336	0.264	0.293	0.193	0.273	0.308	0.330	0.293	0.300	0.220	0.297
Health Promoting Lifestyle (HPL)		0.783	0.826	0.788	0.683	0.706	0.734	0.639	0.537	0.603	0.539	0.531
Self-Actualization (SA)			0.562	0.544	0.550	0.402	0.453	0.576	0.457	0.598	0.522	0.449
Health-Responsibility (HR)				0.552	0.455	0.589	0.528	0.490	0.428	0.416	0.420	0.419
Stress Management (SM)					0.394	0.509	0.556	0.464	0.413	0.431	0.341	0.399
Interpersonal Support (IS)						0.371	0.364	0.488	0.345	0.490	0.458	0.402
Nutrition							0.427	0.457	0.424	0.399	0.367	0.378
Physical Activity (PA)								0.405	0.351	0.381	0.312	0.347
Quality of life (QOL)									0.793	0.863	0.817	0.915
Physical Health (PH)										0.646	0.531	0.596
Psychological State (PS)											0.706	0.659
Social Relationship (SR)												0.659

*Note*: All correlation were significant, *p* < .001

### The mediating effect of Health-promoting lifestyle (HPL)

The relationship between SPH and QoL mediated by the HPL was confirmed. SPH affected HPL (B = 0.201, *p* < .0001), with an explained variation of 14.0%. HPL and SPH affected QoL (B = 0.077 and 0.696, *p* < .0001), with an explained variation of 42.7%. HPL’s total effect, direct effect, and indirect effect were 0.216, 0.077, and 0.140, respectively. All values were significant, indicating that HPL played a partial mediation role between SPH and QoL (Table 4).

**Table 4:** Mediation effect of HPL between SPH and QoL

		HPL						QoL			
		Coeff. (B)	SE		*t*	95%CI		Coeff. (B)	SE	*t*	95%CI
HPL		0.201	0.023		8.705	0.155, 0.246		0.077	0.024	3.204	0.030, 0.123
SPH								0.696	0.043	16.118	0.061, 0.781
Constant		2.062						0.384			
Adjust R2		0.140						0.427			
*p* value		< 0.0001						< 0.0001			
**Relationship**		**Total Effect**		**Direct Effect**		**Indirect Effect**		**Confidence Interval**			
								**Low Bound**		**Upper Bound**	
SPH → HPL→ QoL		0.216 (*p* < .001)		0.077 (*p* = .001)		0.140^*^		0.096		0.188	

*Note*: The work experience and nursing position were controlled as covariates

*p < .05

## Discussion

The results of this study show significant differences in the QoL among nurses across different age groups and work experiences. Nurses aged over 40 tend to have a higher average QoL compared to those aged between 21 and 40. Similarly, this pattern is observed in nurses with more than 12 years of work experience. Both findings suggest that as nurses age and gain experience, the increased familiarity and expertise acquired through clinical work result in improved QoL, consistent with previous research [34, 35]. Furthermore, the positive correlation between nurse quality of life and their position in the healthcare hierarchy can be linked to higher salaries, fixed vacation times, and improved working conditions. These advantages allow nurses to enjoy better QoL by providing financial stability and opportunities for personal growth. On the other hand, marital status and level of education did not significantly affect the quality of life of nurses, which is consistent with previous studies [36, 37].

Results indicated that nurses in this study had a low to average level of SPH. This differs from the study by Orszulak et al. [36], in which the study subjects rated their health between satisfactory and average. This discrepancy may be attributed to the differences in the study population; nurses in Taiwan perceived their health status to be far from optimal, suggesting a need to address health promotion lifestyles among this group.

The Health Promotion Administration of Taiwan’s Ministry of Health and Welfare advises that adults should engage in a minimum of 150 min of moderate physical activity or 75 min of vigorous physical activity each week [38]. Physical activity is an important part of a healthy lifestyle. Although the present study found that nurses were almost often engaged in health promotion behaviors, similar to the findings of previous studies [1, 39], it should be noted that among the six dimensions of HPL, physical activity received the lowest score. Furthermore, interpersonal support received the highest score, consistent with previous studies [40–42]. This may be due to the fact that Chinese-speaking communities have always attached importance to the maintenance of interpersonal relationships [43].

A moderate QoL was reported among nurses in this study, which is consistent with a previous study [36]. Interestingly, out of the four domains of QoL, nurses scored lowest in the physical health domain. Consistent with the results of Samiei Siboni et al. [44], this phenomenon can be explained by the lower frequency of physical activity among nurses in this study, implying the need for improvement.

The study findings revealed that SPH is positively correlated with HPL. Participants with higher subjective health were found to engage in more health-promotion behaviors, thus creating a healthier lifestyle, as demonstrated in other studies [42, 45]. Furthermore, a significant positive correlation was found between HPL and QoL, suggesting individuals who adopt a health-promoting lifestyle tend to have a better quality of life. This finding is consistent with previous studies that have reported a positive association between HPL and QoL [39].

The study confirmed that HPL partially mediated the relationship between SPH and QoL. This mediating effect occurred in two different stages: (1) SPH had a significant positive effect on HPL, and (2) HPL had a significant positive effect on QoL. Thus, it can be inferred that nurses with better subjective health are more likely to actively improve their QoL through health promoting behaviors. In other words, adopting a higher frequency of health promotion behaviors corresponds to better self-perceived health, better health promotion lifestyle, and better quality of life. These findings are consistent with previous studies that have demonstrated the important mediating role of HPL in the relationship between SPH and quality of life [20, 39, 46].

This study demonstrated a significant mediating effect of HPL on the relationship between SPH and QOL among nurses, providing a new perspective on current existing knowledge and information on interventions aimed at the improvement of nurses’ QoL. However, the study possesses several limitations. First, the cross-sectional nature of the study design represents the responses of the sample participants at the time of data collection. Second, the questionnaires used to collect the data carry the risk of all subjective assessments, which may lead to systematic information error. Third, the sample did not take into account environmental factors associated with living in urban or rural areas (e.g., availability of public transportation, fitness centers), as these may affect nurses’ quality of life. A future cohort study could be conducted to investigate the course of change in nursing health behaviors over time, providing more information about the impact of work-related burden on nurses. Furthermore, future studies can explore additional factors related to HPL and its association with SPH and QoL to provide a more comprehensive analysis and enhance the overall effectiveness of a health-promoting lifestyle.

## Conclusions

The study confirmed that HPL has an important role in mediating nurses’ SPH and QoL. Nurse administrators are advised to encourage nurses to monitor their health status and provide health promotion mechanisms to improve their quality of life. Several key strategies are as follows: (1) Prioritize the development of emotional management skills among nurses by organizing periodic workshops or one-on-one sessions with professional mental health counselors. (2) Establish a well-structured shift system tailored to the individual needs to ensure adequate rest for nurses, leaving periods for their physical and psychological recovery. (3) Create a 24/7 fitness facility within the hospital or collaborate with nearby fitness centers to provide discounted memberships, encouraging nurses to exercise during off-duty hours, thus increasing leisure-time physical activity. (4) Address poor eating habits by focusing on the quality of hospital meals and healthy food supplies from nearby establishments. (5) Conduct health education campaigns to improve health literacy among nurses. In general, it is highly recommended for hospitals to introduce an environment conducive to the improvement of nurses’ health-promotion lifestyle.

## Data Availability

The datasets used and analyzed during the current study are available from the corresponding author on reasonable request.

## List of abbreviations

SPH: Self-Perceived Health (SPH)
HPL: Health Promoting Lifestyle
QoL: Quality of Life
WHO: World Health Organization
MOS SF-36: Medical Outcome Study 36-Item Short-Form
HPLP: Health Promoting Lifestyle Profile
